# *Anaplasma* infection of Bactrian camels (*Camelus bactrianus*) and ticks in Xinjiang, China

**DOI:** 10.1186/s13071-015-0931-1

**Published:** 2015-06-10

**Authors:** Youquan Li, Jifei Yang, Ze Chen, Gege Qin, Yaqiong Li, Qian Li, Junlong Liu, Zhijie Liu, Guiquan Guan, Hong Yin, Jianxun Luo, Lin Zhang

**Affiliations:** State Key Laboratory of Veterinary Etiological Biology, Key Laboratory of Veterinary Parasitology of Gansu Province, Lanzhou Veterinary Research Institute, Chinese Academy of Agricultural Sciences, Lanzhou, 730046 P. R. China; Jiangsu Co-innovation Center for Prevention and Control of Important Animal Infectious Diseases and Zoonoses, Yangzhou, 225009 P. R. China

**Keywords:** *Anaplasma platys*, Detection, Bactrian camel, PCR, Xinjiang, China

## Abstract

**Background:**

To date, anaplasmosis has been reported to be a subclinical disease in Indian and Arabian one-humped camels (*Camelus dromedariu*s) and llamas (*Lama glama*). However, no information on *Anaplasma* infection in two-humped Bactrian camels (*Camelus bactrianus*) in China has been published to date. The aim of this study was to investigate the prevalence of *Anaplasma* spp. in domestic Bactrian camels and ticks in Xinjiang, China.

**Findings:**

A total of 382 ticks were collected from the Bactrian camels and from environmental sources. Of these, 84 were morphologically identified as belonging to the *Rhipicephalus sanguineus* group and genetically identified (12S rDNA, 16S rDNA and the cytochrome c oxidase 1 genes) as *R. sanguineus* group ticks (temporally designated as *Rhipicephalus* sp. Xinjiang). PCR testing showed that 7.2 % (20/279) of the camels harbored *Anaplasma platys* DNA. However, microscopic examination revealed no *A. platys* inclusions in blood smears from the camels. The PCR prevalence of *A. platys* DNA was 9.5 % (6/63) in *Rhipicephalus* sp. Xinjiang from the Bactrian camels and 14.3 % (3/21) in *Rhipicephalus* sp. Xinjiang from the vegetation. *A. platys* DNA was not detected by PCR in other tick species (*Hyalomma asiaticum*, *Dermacentor niveus* and *Hyalomma dromedarii*), and no other *Anaplasma* species were detected in these samples.

**Conclusions:**

This is the first report of *A. platys* in Bactrian camels in Xinjiang, China. The moderate positivity observed indicates that these animals might be a natural host for this pathogen in China.

## Background

Anaplasmosis is an infectious disease caused by a Gram-negative obligate intracellular bacterium of the Anaplasmataceae family (order Rickettsiales). The order was reclassified in 2001 and includes several genera, including *Anaplasma*, *Ehrlichia*, *Neorickettsia*, and *Wolbachia* [1]. These arthropod-transmitted bacteria are important emerging pathogens of both animals and humans [2]. Of the known *Anaplasma* spp., *Anaplasma marginale* is the most virulent, and is responsible for extensive economic losses to farmers in tropical and subtropical areas [3–5]. *A. marginale* is considered capable of infecting dromedaries, cervids, domestic buffalos and cattle [6–9]. *A. phagocytophilum* is the causative agent of human granulocytic anaplasmosis [10, 11], a life-threatening disease associated with high mortality rates in humans; *A. phagocytophilum* can also infect dromedaries, llamas, and cervids [7, 12]. *A. platys*, the causative agent of canine infectious cyclic thrombocytopenia, is usually a mild disease with a worldwide distribution, although its virulence may vary from region to region [13, 14]. *A. platys* is supposed to be transmitted by *R. sanguineus* sensu lato [15, 16]. Nonetheless, a definitive proof of the vector competence of this tick for *A. platys* is currently lacking [17].

The genus *Camelus* includes two species: *Camelus dromedariu*s, a dromedary or one-humped camel, and *Camelus bactrianus*, a Bactrian or two-humped camel. Currently, there are around 160,000 Bactrian camels in Xinjiang, China, but dromedary camels are wild and there are no accurate statistics about them [18]. Anaplasmosis in camels is reported to be a subclinical disease in Tunisian, Indian and Arabian one-humped camels [7, 19–24]. To date, only *A. marginale* and *A. phagocytophilum* have been reported to cause anaplasmosis in dromedaries and llamas [7, 19–24]. Information on canine, bovine, ovine, caprine and cervine anaplasmosis has been recorded [25–28], but no information on anaplasmosis in camels in China is available. The aim of this study was to investigate the prevalence of *Anaplasma* species in domestic Bactrian camels and ticks in Xinjiang, China.

## Methods

### Sample collection

The region investigated in Xinjiang, China is located at latitudes 39°30′ to 41°27′ north and longitudes 79°39′ to 82°01′ east. The study was performed in May 2014. In total, 279 blood samples were randomly collected from free-choice grazing camels in desert regions. Blood smears were prepared from blood samples obtained by ear venipuncture of individual Bactrian camels. All of camels were clinically examined before blood sample collection.

Ticks on camels were collected directly and transferred to labeled vials, whereas dragging was used to collect ticks from the vegetation [29]. This method is considered inefficient for sampling *Hyalomma* spp. ticks because they are concealed in favorable micro-habitats and display an active host-seeking behavior [30]. Thus, *Hyalomma* spp. were collected from the walls and crevices of camel sheds. Three hundred and eighty-two ticks were collected from camels, environment vegetation, and animal sheds. All ticks were labeled according to their sources (*i.e.*, camel, vegetation or animal shed), and were morphologically identified according to the methods of Teng and Jiang [31]. Ticks belonging to the *R. sanguineus* group (*n* = 84) were subject to further identification using the methods of Dantas-Torres *et al.* [32]; these methods are based on PCR amplification and sequence analysis of the 12S rDNA, 16S rDNA, and cytochrome c oxidase (*cox1*) genes. PCR products of three genes were purified and ligated into pGEM T easy vector. The recombinant plasmids were transformed into *Escherichia coli* DH5α competent cells. As for each PCR samples, at least three positive clones identified by their own relative specific primers were sequenced by the GenScript Corporation (NJ, China). Basic Local Alignment Search Tool software (http://blast.ncbi.nlm.nih.gov/Blast.cgi?CMD=Web&PAGE_TYPE=BlastHome) was used for sequence analysis. Representative 12S and 16S rDNA genes and *cox1* gene nucleotide sequences have been deposited in GenBank.

### Microscopy

Blood smears were air-dried, fixed in methanol, stained with a 10 % solution of Giemsa (Sigma-Aidrich, Santa Clara, CA, USA) in phosphate-buffered saline (pH 7.2), and then subjected to microscopic examination.

### DNA extraction

After species identification, all the ticks were washed in 70 % ethanol, rinsed three times in sterile phosphate-buffered saline, and dried on filter papers. They were separated individually and stored at −80 **°**C until the DNA extraction. Genomic DNA from 279 whole blood samples and 382 of the tick samples was extracted individually using DNeasy Blood & Tissue Kit (Qiagen, Hilden, Germany) according to the manufacturer’s instructions. DNA yields were determined using a NanoDrop ND-2000 Spectrophotometer (Nanodrop Technologies, Wilmington, DE, USA).

### Molecular detection of *Anaplasma* spp. using species-specific primer sets

PCR was used to detect and identify *Anaplasma* spp. from Bactrian camels and ticks using the genera-specific and species-specific primers listed in Table 1 [16, 33–35]. PCR reactions were performed in a DNA thermocycler (Bio-Rad, Hercules, CA, USA) and the PCR conditions were the same as those reported previously [16, 33–35]. DNA from ovine whole blood without *Anaplasma* and *Ehrlichia* DNA and DNA from each bacterial species were included in each PCR reaction as negative and positive controls; of positive control, *A. platys* DNA originated from dog, *A. ovis* DNA from goat, *A. marginale* DNA from cattle, *A. bovis* DNA and *A. phagocytophilum* DNA from red deer. To assess the presence of specific bands for *Anaplasma* spp., PCR products were separated by 1.5 % agarose gel electrophoresis and the purified DNA products were sequenced. Only when the PCR and sequencing results were consistent were the samples identified as positive for *Anaplasma* spp. Representative sequences of the newly identified pathogens have been deposited in GenBank.

**Table 1:** Sequences of the primers used in this study

Pathogen	Target gene	Primers		Final amplicon size (bp)	References
		Primer name	Oligonucleotide sequences (5′-3′)		
*Anaplasma & Ehrlichia*	16S rRNA	EC9	TACCTTGTTACGACTT	1462	[33]
		EC12A	TGATCCTGGCTCAGAACGAACG		
*A. bovis*	16S rRNA	AB1f	CTCGTAGCTTGCTATGAGAAC	551	[33]
		AB1r	TCTCCCGGACTCCAGTCTG		
*A. phagocytophilum*	16S rRNA	SSAP2f	GCTGAATGTGGGGATAATTTAT	641	[33]
		SSAP2r	ATGGCTGCTTCCTTTCGGTTA		
*A. marginale*	msp4	Amargmsp4 F	CTGAAGGGGGAGTAATGGG	344	[34]
		Amargmsp4 R	GGTAATAGCTGCCAGAGATTCC		
*A. ovis*	msp4	Aovismsp4 F	TGAAGGGAGCGGGGTCATGGG	347	[34]
		Aovismsp4 R	GAGTAATTGCAGCCAGGGACTCT		
*A. platys.*	16S rRNA	PLATYS F	AAGTCGAACGGATTTTTGTC	506	[16]
		PLATYS R	CTTTAACTTACCGAACC		
	groEL	PLA-HS475F	AAGGCGAAAGAAGCAGTCTTA	724	[35]
		PLAT-HS1198R	CATAGTCTGAAGTGGAGGAC		
	*glt*A	PLA-CSM136F	TTGCAAAAAGTAAGCGGAGC	1459	[35]
		PLA-CS1359R	AACCACAGGCTTATGACAAC		

### Ethical approval

This study was approved by the Animal Ethics Committee of the Lanzhou Veterinary Research Institute, CAAS (No. LVRIAEC2013-010). Use of the field samples was approved by the Committee on Animal Research and Ethics of China.

## Findings

### Tick identification

The 382 ticks identified herein included the following four species: *Hyalomma asiaticum* (*n* = 186, 154 adults from camels and 32 adults from animal sheds), *R. sanguineus* group (*n* = 84, 63 adults from camels and 21 adults from vegetation), *Dermacentor niveus* (*n* = 60, 42 adults from camels and 18 adults from vegetation), and *Hyalomma dromedarii* (*n* = 52, 39 adults and 2 nymphs from camels, and 11 adults from animal sheds) (Table 2). Ticks morphologically identified as belonging to the *R. sanguineus* group were also genetically characterized as *R. sanguineus* group ticks and temporally designated as *Rhipicephalus* sp. Xinjiang. The nucleotide sequences reported in this article have been deposited in GenBank (12S rDNA: KR809575-KR809580; 16S rDNA: KR809581-KR809588; *cox1*: KR809589-KR809595). All *R. sanguineus* group ticks isolates in the present study shared 99.8 %–100 % identity in their 12S rDNA gene sequences, 100 % identity in 16S rDNA and *cox1* gene sequences. As compared with sequences available in Genbank, the highest sequence identities were found with sequences labeled as *R. turanicus* from USA. However, compared with a reference sequences of *R. turanicus* from Turkmenistan they showed identity of 96.2-96.5 % for 12S gene (GenBank accession number: KF145151), 95.3 % for 16S gene (GenBank accession number: KF145150) and 90.5 % for *cox1* gene (GenBank accession number: KF145153).

**Table 2:** Prevalence of *Anaplasma* spp. in Bactrian camels (*Camelus bactrianus*) and ticks in Xinjiang, China (2014)

Host	No. of samples	Prevalence of *Anaplasma* spp. by PCR		
		*A. platys*	*A. marginale*	*A. phagocytophilum*
Bactrian camel	279	7.2 % (20/279)	0	0
*Rhipicephalus* sp.^b^	21	14.3 % (3/21)	0	0
*Rhipicephalus* sp.^c^	63	9.5 % (6/63)	0	0
*Hy. asiaticum* ^d^	32	0	0	0
*Hy. asiaticum* ^c^	154	0	0	0
*D. niveus* ^b^	18	0	0	0
*D. niveus* ^c^	42	0	0	0
*Hy. dromedarii* ^d^	11	0	0	0
*Hy. dromedarii* ^a,c^	41	0	0	0

^a^ *Hyalomma dromedarii* (41 ticks): 39 adult ticks and 2 nymph ticks

^b^Ticks collected from vegetation

^c^Ticks collected from Bactrian camels

^d^Ticks collected from walls and crevices of animal sheds

### Microscopic examination of blood smears

No obvious suspected cases of anaplasmosis were observed based on the signs of fever, anemia, emaciation, slight ataxia, and anorexia in the geographical region we investigated. No microscopic evidence of *Anaplasma* infections in the blood smears from the Bactrian camels was observed.

### PCR detection of *Anaplasma* using species-specific primer sets

The nearly full-length 16S rRNA gene sequences for *A. platys* were 1456 bp using EC9/EC12A primers, which are specific for *Anaplasma* and *Ehrlichia* spp. The accession numbers of the 16S rRNA gene sequences are KM246797-KM246800 and KP939254-KP939256 (*Anaplasma*). For the 16S rRNA gene, PCR products of 506 bp were obtained for *A. platys* using the species-specific PLATYS F and PLATYS R primers (GenBank accession numbers: KP939260-KP939262). For the groEL gene, PCR products of 724 bp were obtained for *A. platys* using species-specific PLA-HS475F and PLA-HS1198R primers (GenBank accession numbers: KR011925 and KR011926). For the *glt*A gene, PCR products of 1459 bp were obtained for *A. platys* using species-specific PLA-CSM136F and PLA-CS1359R primers (GenBank accession numbers: KR011927 and KR011928). The prevalence of *A. platys* in Bactrian camels and ticks was estimated from the number of PCR-positive samples based on the 16S rDNA gene, the groEL gene and the gltA gene, and the three species-specific primer sets produced results that were consistent for the prevalence of *A. platys* in Bactrian camels and ticks. The PCR data revealed that the prevalence of *A. platys* in camels was 7.2 % (20/279). The prevalence of *A. platys* in *Rhipicephalus* sp. Xinjiang collected from Bactrian camels was 9.5 % (6/63), while in *Rhipicephalus* sp. Xinjiang from the environment was 14.3 % (3/21) (Table 2). *A. platys* DNA was not detected in *Hy. asiaticum* (*n* = 186), *D. niveus* (*n* = 60), and *Hy. dromedarii* (*n* = 52). Other *Anaplasma* spp. (*A. phagocytophilum*, *A. marginale, A. bovis* and *A. ovis*) were not detected, neither in camels nor in ticks.

## Discussion

To the best of our knowledge, this study is the first to report on the prevalence of *Anaplasma* infection Bactrian camel in China. The three species-specific primer sets for *A. platys* (based on the 16S rDNA gene, the groEL gene and the *glt*A gene) gave consistent PCR results, confirming the occurrence of *A. platys* in Bactrian camels and ticks. The highest prevalence of *A. platys* occurred in *Rhipicephalus* sp. Xinjiang ticks collected from the environment and in *Rhipicephalus* sp. Xinjiang from Bactrian camels; these camels had the lowest prevalence of *A. platys* infection. As far as we know, this is the first report of *A. platys* infection of Bactrian camels worldwide. To date, two other *Anaplasma* spp., *A. marginale* and *A. phagocytophilum*, have been associated with anaplasmosis in dromedaries and llamas [7, 19–22]. However, *A. marginale* and *A. phagocytophilum* were not detected in the Bactrian camels and four species of ticks that we studied in Xinjiang, China. Other *Anaplasma* spp., such as *A. ovis* and *A. bovis*, were also not detected in our samples. Anaplasmosis is reported to be a subclinical disease in Tunisian, Indian, and Arabian one-humped camels [20–24]. In the present study, *A. platys* infections were detected by PCR but there were no obvious signs of anaplasmosis (fever, progressive anemia, generalized lymph node enlargement, emaciation, slight ataxia and anorexia) in the Bactrian camels. One possible explanation for this finding is that *A. platys* infections in camels show only minimal or no subclinical signs. Dromedary camels are wildlife in Xinjiang, but samples from these animals were not collected in this study; hence, information on anaplasmosis in dromedary camels is not available.

*A. platys* infects mainly dogs, and cases of canine anaplasmosis have been reported in many countries [13–17, 25, 35]. *A. platys* infections have been reported in dogs in southern China [25], but the tick species that transmit it have not been determined as yet. Recently, an infection with *A. platys* was detected in a cat in Brazil [36], and infections with this bacterium have also been detected in goats [26] and red deer in China [28]. DNA from *A. platys* was also detected in red foxes [37], in a veterinarian with clinical anaplasmosis [38], and in two women from Venezuela [39]. These data indicate that *A. platys* bacteria have a broad host range. However, the ability of *A. platys* to act as a zoonotic pathogen has not been established; hence, further studies are necessary to determine its zoonotic potential.

Detection of *A. platys* in the moderate number of the Bactrian camels sampled herein indicates that these animals are exposed to the bacterium and that a desert life cycle for this pathogen is possible for camel populations in Xinjiang, China. Therefore, Bactrian camels might play a role in the transmission of this pathogen, possibly by serving as natural hosts. Additionally, because *A. platys* infections have been reported previously in dogs, we speculate that, in Xinjiang, *A. platys* infections in Bactrian camels might be transmitted from ticks fed on naturally infected dogs. In the desert region of Xinjiang, the animals that cohabitate with Bactrian camels include dogs, wolves, foxes and rabbits. Dogs are a natural host of *A. platys*. However, additional studies will be needed to determine whether wolves, foxes and rabbits can be infected by *A. platys*. Furthermore, PCR detected DNA from *A. platys* in *Rhipicephalus* sp. Xinjiang ticks collected from Bactrian camels and in local vegetation. Several studies have reported the presence of *A. platys* DNA in *R. sanguineus* group ticks; hence, this pathogen is supposed to be transmitted by *R. sanguineus* group ticks [15, 16, 40]. The finding of *A. platys* DNA in *Rhipicephalus* sp. Xinjiang ticks raises important questions regarding their role as vectors of *A. platys* for camels in Xinjiang, China.

## Conclusion

This is the first report to demonstrate the occurrence of *A. platys* in Bactrian camels in Xinjiang, China. The moderate prevalence of *A. platys* we observed in Bactrian camels indicates that they might be a host for this pathogen in desert regions.
